# Herpes Zoster Presenting As Cutaneous Vasculitis in the Upper Extremity

**DOI:** 10.7759/cureus.20391

**Published:** 2021-12-13

**Authors:** Jacob H Nelson, Chong Foo, Lauren Hammock, Olivia Lucero

**Affiliations:** 1 Dermatology, Western University of Health Sciences, Lebanon, USA; 2 Dermatology, Peace Health, Eugene, USA; 3 Pathology, Pathology Consultants, Eugene, USA; 4 Dermatology, Oregon Health & Science University, Portland, USA

**Keywords:** herpes zoster virus, cutaneous vasculitis, vzv, lcv, varicella-zoster virus, palpable purpura, dermatology case report

## Abstract

The varicella-zoster virus (VZV), a member of the Herpesviridae family (HHV-3), is the pathogen responsible for causing herpes zoster, the skin eruption known as shingles. This report describes a rare presentation of herpes zoster involving cutaneous vasculitis in the unilateral upper extremity in an immunocompetent patient. Histologic evaluation confirmed a diagnosis of leukocytoclastic vasculitis and yielded a positive VZV immunoperoxidase stain. An approach to histologic evaluation of this case is discussed.

## Introduction

Herpes zoster is a viral skin infection caused by a reactivation of latent varicella-zoster virus (VZV) and typically presents as a painful vesicular eruption of the skin in a dermatomal distribution [1,2]. Cutaneous small-vessel vasculitis is defined as neutrophilic inflammation within the wall of post-capillary venules in the dermis [3]. Clinically, it presents as palpable purpura and may have a variety of etiologies, including the spread of infection to the blood vessel endothelium [3]. We present a case of cutaneous vasculitis secondary to herpes zoster in a patient without any known immunodeficiencies.

## Case presentation

A 60-year-old Caucasian male was referred to the dermatology clinic for evaluation of a painful rash. Four weeks prior, he noticed a “bug bite” on his left wrist. Over the following 24 hours, he experienced a red and purple eruption on the left forearm, elbow, and left dorsal hand accompanied by a tingling sensation and edema of the affected skin. After several days, he sought care from an urgent care clinic due to discomfort and was prescribed a regimen of cephalexin. He failed to improve and thus presented to his primary care physician and was started on a prednisone taper (40mg PO daily for four days, then 20mg PO daily for four days, then 10mg PO daily for four days) and referred to the dermatology clinic. 

Upon presenting to the dermatology clinic, he had completed six days of the prednisone taper, which led to mild improvement of his symptoms.

The patient denied any palliating or provoking factors, previous skin disease, or sick contacts. He endorsed a history of varicella infection during childhood and denied receiving the shingles vaccine. A review of systems revealed the absence of systemic symptoms such as malaise, fever, muscle pain, and joint pain. His medication regimen includes tamsulosin for benign prostatic hypertrophy and intermittent Vicodin and ibuprofen for chronic low back pain. The patient also denied any known immunodeficiencies. The patient drinks alcohol occasionally and has a pack-year history of cigarette smoking and marijuana use.

**Figure 1:**
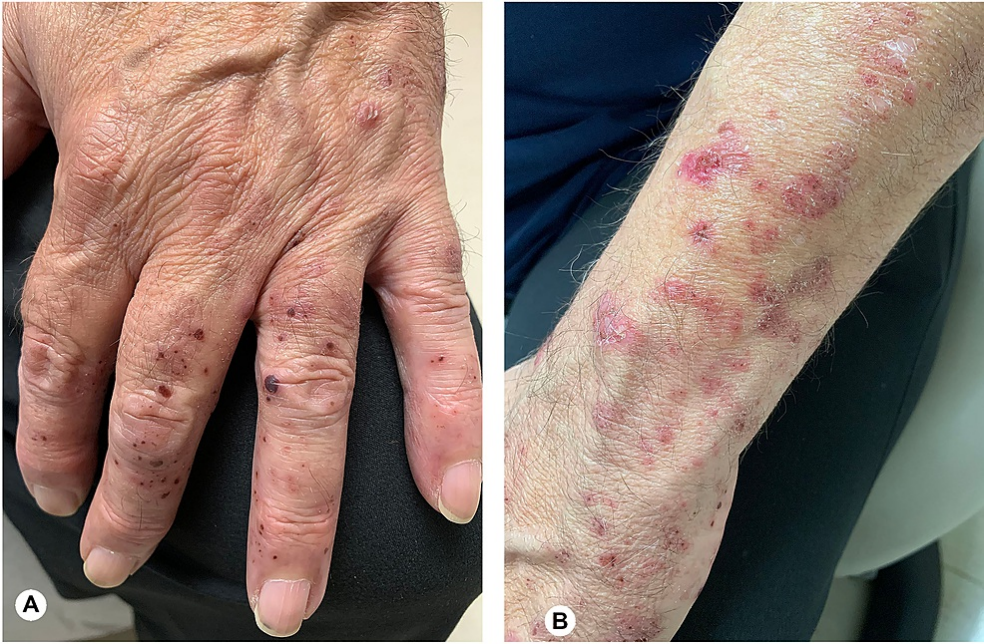
Photographs showing purpuric papules involving left hand and forearm. (A) Patient’s left hand showing violaceous purpuric papules involving the dorsal aspect of the left fifth through second digits. (B) Patient’s left wrist showing pink irregular papules.

Physical exam revealed normal mood and affect, and orientation to place, person, and time. Full-body skin exam revealed pink ill-defined irregular papules of varying size on the left distal forearm, and well-defined, round, violaceous papules on the dorsal and palmar aspects of the left digits two through five. There were no intact vesicles present on the gross exam (Figures 1A, 1B).

**Figure 2:**
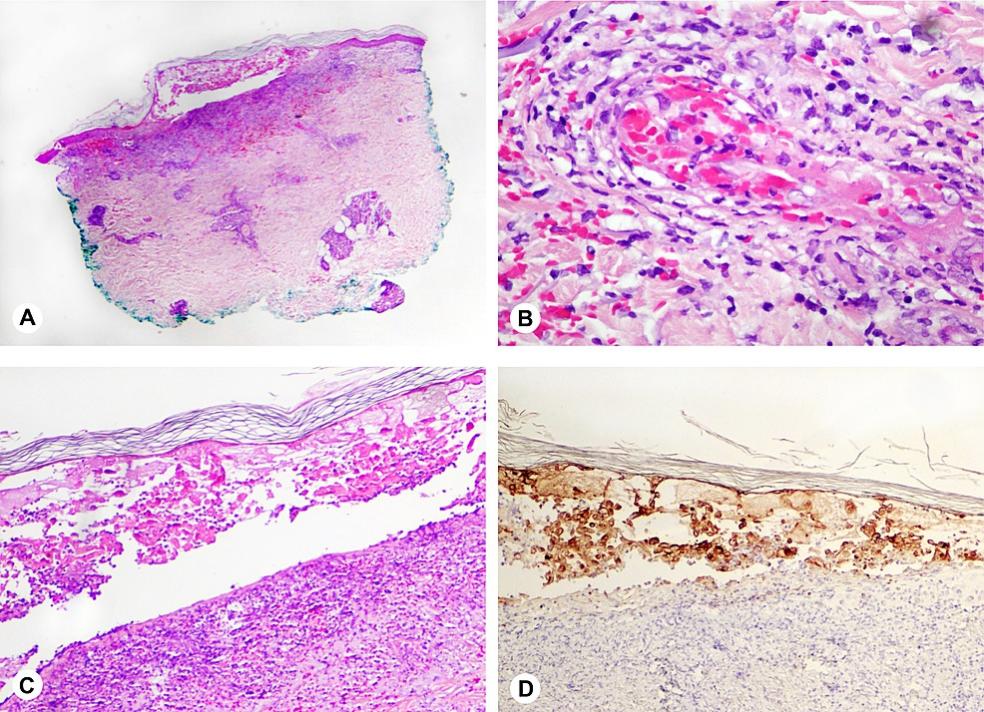
Examination of biopsies taken from the patient’s left upper extremity (H&E). (A) A blister with numerous necrotic keratinocytes and a superficial and deep neutrophilic infiltrate with the destruction of vessels (20x). (B) A small vessel with marginating neutrophils, fibrin thrombus, fibrinoid necrosis of vessel wall, karyorrhectic debris, and numerous extravasated red cells (400x). (C) Necrotic keratinocytes with marked acantholysis and large red intranuclear inclusions (100x). (D) Strongly positive immunostaining for varicella-zoster antigen (100x).

Two punch biopsies were performed on the left proximal and distal upper extremity and sent for histological evaluation. Both sites displayed similar findings and stated that the specimen from the distal biopsy site appeared more evolved. Findings included small vessels with marginating neutrophils, fibrin thrombi, fibrinoid necrosis of blood vessel walls, karyorrhectic debris, and numerous extravasated red cells. Pathology also reported a vesicle with numerous necrotic keratinocytes with marked acantholysis and large red intranuclear inclusions, and a superficial and deep neutrophilic infiltrate with the destruction of vessels. The tissue samples at both sites also stained positive for VZV antigen and stained negative for herpes simplex virus (HSV) antigen. The findings at both biopsy sites were consistent with a diagnosis of leukocytoclastic vasculitis (LCV) in the setting of herpes zoster infection (Figures 2A-2D).

The patient agreed to valacyclovir 1,000mg three times daily for seven days. He was advised to continue the prednisone taper prescribed by the primary care provider of 10mg daily for four additional days. The patient currently reports that his symptoms have been steadily improving with the prescribed therapies.

## Discussion

There are only a small number of reports describing herpes zoster presenting as a small vessel vasculitis. Two case reports describe immunosuppressed patients with cutaneous vasculitis in the lower extremity in patients being treated for T-cell lymphoma and systemic sarcoidosis [4,5]. The diagnosis of herpes zoster was confirmed by viral culture and immunohistochemistry, respectively. We found only one report describing an immunocompetent patient with small vessel vasculitis, also in the lower extremity, with the presence of VZV confirmed via polymerase chain reaction (PCR) [6]. To our knowledge, this case represents the fourth case to be added to the literature where VZV presents without the classic vesicular eruption, but rather manifesting as LCV. To our knowledge, it is the only case describing it in the upper extremity.

Our differential diagnosis included other small vessel vasculidities such as granulomatosis with polyangiitis, idiopathic thrombocytopenic purpura (ITP), and drug reaction. Our patient denied any bleeding or systemic symptoms and denied taking any new medications or supplements. There are, in fact, reports of medications such as nonsteroidal anti-inflammatory drugs (NSAIDs) causing drug-induced LCV. [7] Ultimately herpes zoster was diagnosed by histopathologic studies.

This case displayed interesting histologic findings. Figure 2B displays classic findings of LCV and Figure 2C depicts classic histologic findings of the Herpesviridae virus infections. Because infection by HSV and VZV are indistinguishable on histology, immunohistochemistry or other special testing is required to identify the causative pathogen [8]. Immunoperoxidase stain is commonly employed to differentiate between HSV and VZV infections by incubating a specimen slide in a bath of anti-VZV or anti-HSV antibody linked to a chromophore that is activated by peroxidase enzyme after the antibody has bound the viral antigen [9].

## Conclusions

In summary, we report a highly unusual case of herpes zoster presenting as cutaneous vasculitis in the upper extremity. The literature describing VZV-induced vasculitis is very sparse and not well defined. Our case is made more unusual by the presentation occurring in a patient without any known immunodeficiencies. The diagnosis was confirmed by findings of LCV on histology and positive VZV-specific immunoperoxidase staining. The patient was successfully treated with a regimen of valacyclovir and prednisone.
